# Clonality of circulating tumor cells in breast cancer brain metastasis patients

**DOI:** 10.1186/s13058-019-1184-2

**Published:** 2019-09-03

**Authors:** Carlotta Riebensahm, Simon A. Joosse, Malte Mohme, Annkathrin Hanssen, Jakob Matschke, Yvonne Goy, Isabell Witzel, Katrin Lamszus, Jolanthe Kropidlowski, Cordula Petersen, Anja Kolb-Kokocinski, Sascha Sauer, Kerstin Borgmann, Markus Glatzel, Volkmar Müller, Manfred Westphal, Sabine Riethdorf, Klaus Pantel, Harriet Wikman

**Affiliations:** 1 Department of Tumor Biology, University Medical Center Hamburg-Eppendorf, Hamburg, Germany; 2 Department of Neurosurgery, University Medical Center Hamburg-Eppendorf, Hamburg, Germany; 3 Department of Gynecology, University Medical Center Hamburg-Eppendorf, Hamburg, Germany; 4 Department of Neuropathology, University Medical Center Hamburg-Eppendorf, Hamburg, Germany; 5 Department of Radiotherapy and Radiooncology, University Medical Center Hamburg-Eppendorf, Hamburg, Germany; 6 Laboratory of Radiobiology and Experimental Radiooncology, University Medical Center Hamburg-Eppendorf, Hamburg, Germany; 7 Wellcome Sanger Institute, Hinxton, Cambridge, CB10 1SA UK; 8 Max Delbrück Center for Molecular Medicine (BIMSB and BIH), Berlin, Germany; 9 Max Planck Institute for Molecular Genetics, Otto Warburg Laboratory, Berlin, Germany

**Keywords:** CTC, Breast cancer, Brain metastases, Clonality, CNA, NGS, Chromosomal aberrations

## Abstract

**Background:**

The incidence of brain metastases in breast cancer (BCBM) patients is increasing. These patients have a very poor prognosis, and therefore, identification of blood-based biomarkers, such as circulating tumor cells (CTCs), and understanding the genomic heterogeneity could help to personalize treatment options.

**Methods:**

Both EpCAM-dependent (CellSearch® System) and EpCAM-independent Ficoll-based density centrifugation methods were used to detect CTCs from 57 BCBM patients. DNA from individual CTCs and corresponding primary tumors and brain metastases were analyzed by next-generation sequencing (NGS) in order to evaluate copy number aberrations and single nucleotide variations (SNVs).

**Results:**

CTCs were detected after EpCAM-dependent enrichment in 47.7% of the patients (≥ 5 CTCs/7.5 ml blood in 20.5%). The CTC count was associated with ERBB2 status (*p* = 0.029) of the primary tumor as well as with the prevalence of bone metastases (*p* = 0.021). EpCAM-independent enrichment revealed CTCs in 32.6% of the patients, especially among triple-negative breast cancer (TNBC) patients (70.0%). A positive CTC status after enrichment of either method was significantly associated with decreased overall survival time (*p* < 0.05). Combining the results of both enrichment methods, 63.6% of the patients were classified as CTC positive. In three patients, the matched tumor tissue and single CTCs were analyzed by NGS showing chromosomal aberrations with a high genomic clonality and mutations in pathways potentially important in brain metastasis formation.

**Conclusion:**

The detection of CTCs, regardless of the enrichment method, is of prognostic relevance in BCBM patients and in combination with molecular analysis of CTCs can help defining patients with higher risk of early relapse and suitability for targeted treatment.

**Electronic supplementary material:**

The online version of this article (10.1186/s13058-019-1184-2) contains supplementary material, which is available to authorized users.

## Background

Breast cancer represents the most frequent malignancy among women worldwide. Due to the development of new therapeutic options, the life expectancy of breast cancer patients has increased steadily. Nevertheless, breast cancer still constitutes one of the main causes of death in young women in western countries and the overwhelming majority of deaths are due to metastases [1, 2]. Breast cancer brain metastasis (BCBM), which occurs in approximately 20–40% of patients with metastatic breast cancer, is associated with high morbidity and poor prognosis. The incidence appears to be increasing over the last years, possibly as a result of better therapeutic options for the primary tumors and extracranial metastases [3]. Even after intensive multimodal therapy including resection and radiotherapy, brain metastases are correlated with a poor prognosis, consisting of a median survival time from diagnosis between 4 and 24 months [4, 5]. For this reason, novel and improved therapeutic approaches for BCBM patients are urgently needed. The molecular mechanisms leading to BCBM formation are still incompletely understood. The development of new therapeutic approaches requires, however, detailed understanding of BCBM formation and identification of key drivers of this process. Studies investigating molecular and genetic deviations, which are linked to BCBM, hold promise for prevention of high-risk breast cancer patients for developing brain metastases [3]. We and others have shown that brain metastases harbor additional new aberrations that could not be found in the corresponding primary tumors, highlighting the importance of investigating metastatic cells instead of the primary tumor only [6–8].

Circulating tumor cells (CTCs) have gained much attention, as they are essential for metastasis formation having both prognostic and predictive value in both primary and metastatic breast cancer [9]. CTCs represent a diagnostic source which can be obtained minimal-invasively. Having already undergone several steps needed for successful metastasis, further molecular characterization of these cells may be used as an aid in treatment planning and help to understand the molecular mechanism behind brain metastasis formation [10].

In the current study, we assessed the presence of CTCs in BCBM patients by two different enrichment approaches and characterized both CTCs, primary tumors, and corresponding brain metastases by next-generation sequencing (NGS) in order to investigate the molecular alterations associated with BCBM and its genomic progression towards metastasis.

## Material and methods

### Patient material

This study was performed in accordance with the Declaration of Helsinki. Fifty-seven BCBM patients were enrolled into the study after informed consent was obtained (Ethics Committee of the Medical Board Hamburg approval reference number PV3779). Two tubes of 7.5 ml blood were collected per patient prior to clinical intervention: 38/57 before surgical resection for brain metastasis and 19/57 prior to chemo- or radiotherapy. Thirty-two (56%) patients were diagnosed with an ERBB2-positive primary breast tumor, ten (17.5%) with triple-negative breast cancer (TNBC), and nine (16%) with a hormone receptor (HR) positive tumor. From six cases, the ERBB2 status of the primary tumor was not recorded. The average time between primary diagnosis and diagnosis of brain metastases was 49 months (range 0–180 months). Thirty-seven (64.9%) of the patients showed multiple metastases, whereas 18 (31.6%) of the patients had solitary brain metastases. Median follow-up was 19 months (range 0–113).

### Circulating tumor cell (CTC) detection

In order to obtain CTCs from blood, two enrichment methods were employed. The EpCAM-independent assay was performed as described before [11]. Per patient, 7.5 ml peripheral blood was collected in an EDTA tube and transferred on Ficoll (GE Healthcare). The mononuclear cell fraction was obtained by centrifugation at 1400 rpm for 20 min at 4 °C with brakes off. The cells were resuspended in 50 ml PBS and spun down on cytospins (500,000 cells per slide). If necessary, erythrocyte-lysis (Whole Blood Erythrocyte Lysing Kit, R&D Method Systems, Minneapolis) was performed for 3 min before. Cytospins were dried overnight and stored at − 80 °C until use. CTCs were detected by multicolor immunofluorescence (IF) staining using a combination of antibodies targeting breast cancer and epithelial cell-specific markers. For ERBB2- and hormone receptor-positive patients, a cocktail of keratin antibodies (1:80 AE1/AE3 eFluor570, eBioscience, San Diego, California; A45 Cy3, Micromet, Munich) was used in combination with DAPI and an ERBB2 antibody (CellSearch). CTCs from TNBC patients were detected using the same keratin antibody cocktail, DAPI, and an EGFR antibody (1:50 Biotin, Thermo Scientific, Waltham, Massachusetts). CD45 antibody (1:150 Alexa Fluor 647, BioLegend, San Diego) was used as an exclusion marker for leukocytes. Slides with cells were manually analyzed with a fluorescence-microscope (Zeiss, AxioVision, Jena). In parallel, a second 7.5-ml blood sample from each patient was collected in CellSave collection tubes to be analyzed by the CellSearch® System as previously described [12]. CTCs captured by EpCAM antibodies were detected by antibodies against keratins 8, 18, and 19. DAPI was used to stain nuclear material and antibody against CD45 for negative depletion, excluding leukocytes.

### Whole genome amplification (WGA) and quality control of CTCs

Single CTCs were picked by micromanipulation (micro injector CellTramVario and micromanipulator TransferManNKII, Eppendorf Instruments, Hamburg, Germany) from three different patients. The genomes of the picked cells were amplified by whole genome amplification (WGA) using the MDA-PCR PicoPlex WGA kit for single cells (New England Biolabs, E2620L) as described before [13]. A quality control of the WGA product was performed by a multiplex PCR of the *GAPDH* gene using primers to amplify products of 100, 200, 300, and 400 bp fragments [14]. The PCR products were analyzed using a 2% agarose TAE gel, and samples producing three or four bands were chosen for the NGS analyses. As a positive control, human leukocyte DNA was used.

### DNA extraction from tumor samples

From all three cases of whom CTCs were subjected to WGA, the tissue of the corresponding primary tumor was available. In one of these cases, the tissue of the corresponding brain metastasis was also available. DNA was isolated from formalin-fixed, paraffin-embedded (FFPE) tissue blocks using macrodissection in order to achieve a minimum of 70% tumor cells in the sample. DNA was extracted using the InnuPREP DNA Microkit according to the manufacturer’s protocol (Analytik Jena, Jena, Germany).

### Next-generation sequencing

DNA from the tumor tissues and amplified DNA from single cells underwent whole exome sequencing using the Illumina HiSeq2000 platform. SNV and CNA data analyses were performed using our custom single-cell NGS pipeline as described elsewhere [13]. Briefly, Control-FREEC was employed to evaluate the copy number aberrations with a window size of 500 kb [15]. Copy numbers 2 or 3 were considered “unchanged” in samples with diploid and triploid genomes, respectively; fewer copy numbers were classified as “loss” and more as “gain.” Genetic variant annotation and functional effect prediction was performed using SnpEff [16]. NGS data can be accessed via the European Nucleotide Archive (https://www.ebi.ac.uk/ena) under accession number EGAD00001005020.

### ARID1A immunohistochemical analysis

Tissue microarrays (TMA) with 132 surgical tissue specimens from histologically proven breast cancer brain metastases [7] were used for immunohistochemistry (IHC) staining of ARID1A protein. TMA slides were de-paraffinized in xylol and rehydrated by decreasing ethanol series followed by antigen unmasking by boiling in citrate buffer for 5 min at 120 °C (Citra Plus, BioGenex). Primary mouse monoclonal ARID1A antibody was diluted 1:500 (sc-32761, Santa Cruz Biotechnology) in Dako REAL Antibody Diluent (Agilent Technologies), and the TMA slide was incubated at 4 °C overnight. For visualization, Dako REAL Detection System was used (K5001, Agilent Technologies). Signal intensity (grading 0–3) and signal distribution (percentage of stained cells) were multiplied to a final value and grouped according to the final value into negative (0–0.5), intermediate (0.6–2), and strong (≥ 2) staining.

### Statistical analyses

The CellSearch® System has been cleared by the FDA for CTC analysis in metastatic breast cancer patients using a cutoff of ≥ 5 CTC for positivity [17]. Due to the low frequency of CTCs identified by the EpCAM-independent method, a cutoff of ≥ 1 CTC was used. Statistical analyses were performed using R version 3.5.1 (R Foundation for Statistical Computing) and In-Silico Online, version 2.0 [18]. The *G* test with Williams’ correction or two-tailed *T* test was employed to identify group differences and associations between investigated variables and clinico-histopathological risk factors. Kaplan-Meier estimates with the log-rank test were used to analyze survival differences between the groups. Cox proportional hazard function was used for multivariable analyses. Unsupervised complete hierarchical clustering was employed using Euclidian distance on the chromosomal aberration status gain, loss, and unchanged as determined by Control-FREEC. An alpha level of 0.05 was employed to call statistical significance.

## Results

### CTC detection in blood samples from BCBM patients

**Fig. 1:**
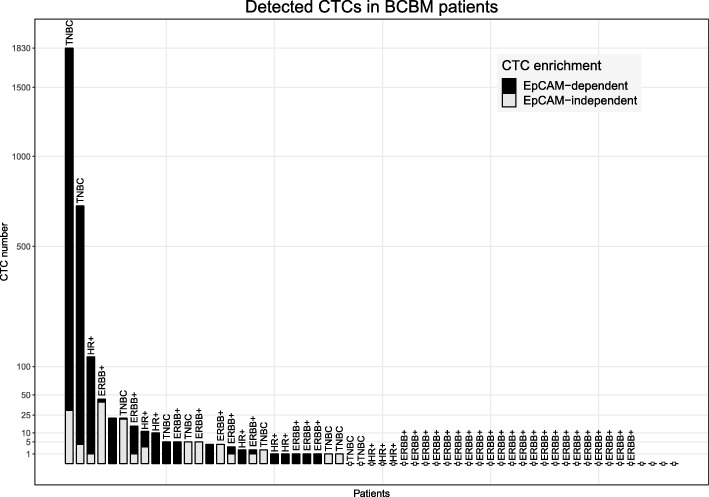
Number of detected CTCs. Bar chart showing the number of CTCs detected after EpCAM-dependent enrichment (gray) and EpCAM-independent enrichment (black) per patient along with their histological subtype. TNBC triple-negative breast cancer, HR+ hormone receptor positive, ERBB+ overexpression of ERBB2

CellSearch results were obtained from 44 patients. Using this EpCAM-dependent method, ≥ 5 CTCs/7.5 ml blood were found in 9 patients (20.5%) and 1–4 CTCs/7.5 ml blood were found in 12 patients (27.3%) (Fig. 1); the median number of CTCs detected was 4 CTCs/7.5 ml blood (range 1–1800). In parallel, the EpCAM-independent method based on Ficoll density gradient centrifugation was used to isolate CTCs according to their physical properties from blood samples of 46 patients. All cases were investigated for keratins and CD45. In addition, EGFR and ERBB2 expression were assessed in the TNBC cases and the ERBB2-positive and hormone receptor (HR)-positive cases, respectively. Altogether, 15 patients (32.6%) had detectable CTCs with a median number of three CTCs per 7.5 ml (range 1–40) (Table 1). In 60% of the TNBC patients, the CTCs were positive for both EGFR and keratins, whereas in 40% the CTC were positive for keratins only. Among the ERBB2-positive cases, a very heterogeneous ERBB2 expression was seen: in one patient, one CTC was detected by ERBB2 expression while being negative for keratins; of two cases, all CTCs were positive for keratins but negative for ERBB2; two patients had keratin and ERBB2-positive CTCs only; one case had five CTCs that were positive for keratin, but only two of them were also positive for ERBB2; finally, in two patients with an ERBB2-positive primary tumor, the single detected keratin-positive CTCs were found to be negative for ERBB2.

**Table 1:** Clinical characteristics. The number of patients tabulated on CTC status and their clinical characteristics. The CTC status was considered positive if ≥ 5 or ≥ 1 CTCs were detected after EpCAM-dependent or EpCAM-independent enrichment, respectively. *p* values were calculated using the *G* test

	≥ 5 CTCs (EpCAM dependent)				≥ 1 CTC (EpCAM independent)			
	Total (*n*)	Neg (*n*)	Pos (*n*)	*p* value	Total (*n*)	Neg (*n*)	Pos (*n*)	*p* value
Total	44	35	9		46	31	15	
Histology								
Ductal	24	20	4	0.655	25	17	8	0.552
Lobular	4	3	1		5	3	2	
Others	2	1	1		3	1	2	
n.a.	14				13			
Age at BM								
< Mean	21	15	6	0.213	25	18	7	0.476
> Mean	23	20	3		21	13	8	
Hormone								
Neg	21	17	4	0.878	20	9	11	0.018
Receptor								
Pos	19	15	4		21	17	4	
n.a.	4				5			
ERBB2								
Neg	16	10	6	0.029	15	6	9	0.021
Pos	24	22	2		26	20	6	
n.a.	4				5			
Subtype								
HR-pos	8	5	3	0.102	5	3	2	0.044
TNBC	8	5	3		10	3	7	
ERBB2-pos	24	22	2		26	20	6	
n.a.	4				5			
Brain surgery								
No OP	14	10	4	0.388	15	9	6	0.471
OP	30	25	5		20	22	9	
n.a.	0				11			
Oligo brain met.								
Multiple	30	24	6	0.827	28	16	12	0.080
Oligo	13	10	3		17	14	3	
n.a.	1				1			
Bone met.								
No	25	23	2	0.021	25	18	7	0.476
Yes	19	12	7		21	13	8	
Liver met.								
No	33	28	5	0.165	31	22	9	0.471
Yes	11	7	4		15	9	6	
Pulmonary met.								
No	36	29	7	0.743	38	28	10	0.064
Yes	8	6	2		8	3	5	

*BM* brain metastasis, *OP* operation, *HR* hormone receptor, *TNBC* triple-negative breast cancer, *met*. metastasis, *n.a*. data not available

In total, 33 cases were analyzed by both the EpCAM-dependent and EpCAM-independent methods. Based on either one of the enrichment methods, 45.6% of the patients had at least one detectable CTC (Fig. 1). Comparing the two techniques revealed a fair agreement (Cohen’s kappa 0.34, *p* = 0.0249), 23.5% and 8.8% of the patients were solely positive for CTCs according to the EpCAM-independent and EpCAM-dependent method, respectively. However, no correlation could be found between the number of CTCs detected after EpCAM-dependent enrichment and those after EpCAM-independent enrichment (tau = 0.0875, *p* = 0.6532), indicating that different CTC populations were detected through the different enrichment techniques.

### Clinical value of detected CTCs

**Fig. 2:**
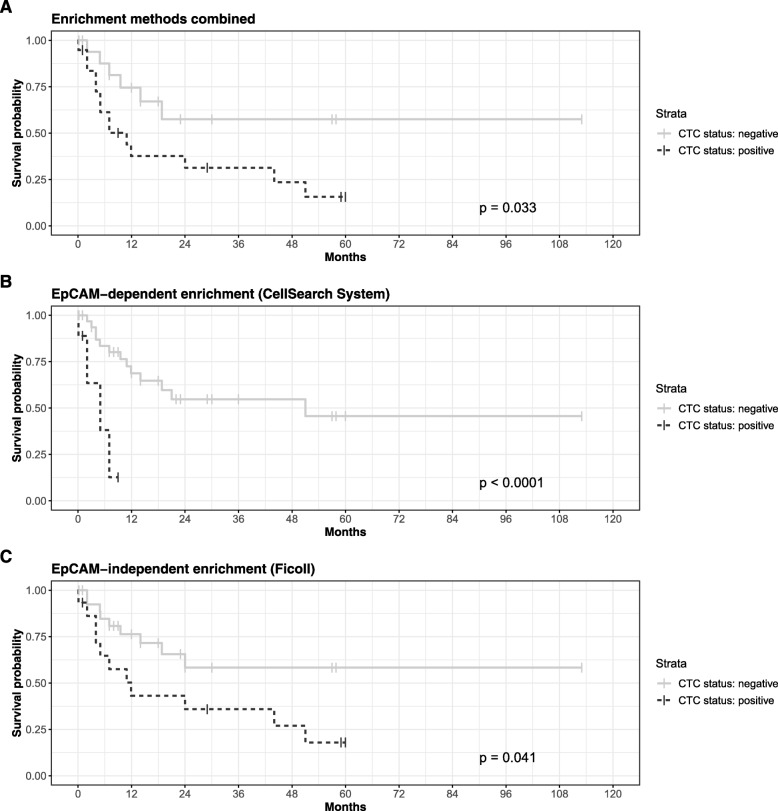
Survival analyses. Kaplan-Meier estimates for patients without (gray solid lines) and with (black dashed lines) detectable CTCs according to the combination of enrichment techniques (**a**), EpCAM-dependent enrichment only (**b**), and EpCAM-independent enrichment only (**c**)

No association could be observed between the detection of CTCs and age or tumor histology (Table 1). In relation to different breast cancer histological subtypes, CTCs detected by the EpCAM-independent method were more common in patients with HR-negative, as well as ERBB2-negative breast cancers as compared to patients with HR-positive or ERBB2-positive breast cancer, respectively (*p* < 0.05, *G* test). Thus, CTCs detected after enrichment by the EpCAM-independent method were most commonly found among TNBC patients (7/41), followed by ERBB2-positive cases (2/41) and HR-positive cases (6/41) (*p* = 0.044, *G* test). In contrast, the EpCAM-dependent method detected more CTCs among ERBB2-negative cases. Furthermore, based on the results obtained by EpCAM-dependent CTC enrichment, patients with additional bone metastases showed more CTCs (7/44) than patients without bone metastases (2/44) (*p* = 0.021, *G* test) (Table 1). However, no interaction between breast cancer subtype and site of metastasis was detected (Table 2, *p* > 0.1, binomial logistic regression model). These results further support the hypothesis that different isolation techniques different populations of CTCs may be detected. Correlating the patient’s follow-up with the results obtained using both CTC enrichment methods, it could be shown that survival after brain metastasis diagnosis was significantly associated with the presence of CTCs (*p* = 0.033, log-rank test, Fig. 2a). Using a threshold of ≥ 5 CTCs on the results obtained after EpCAM-dependent enrichment, a significantly shorter overall survival time among patients with detectable CTCs could be seen (*p* < 0.001, log-rank test, Fig. 2b). In a multivariable analysis on the histological subtype and sites of metastases, the presence of CTCs was significantly correlated with a hazard ratio of 7.19 (95% CI [1.73, 29.95], *p* = 0.0067). Similar results were obtained after EpCAM-independent enrichment, with death confirmed in 73.3% of CTC-positive and 36.7% of the CTC-negative patients (*p* = 0.041, log-rank test, Fig. 2b), but which was not significant in multivariable analysis.

**Table 2:** Breast cancer subtype vs metastasis site. Cross table showing the number of cases of the bone, liver, and pulmonary metastases found per histological subtype of the primary tumor

	Bone met.	Liver met.	Pulmonary met.
HR-pos	7	2	2
ERBB2-pos	3	3	2
TNBC	14	10	5

*HR* hormone receptor, *TNBC* triple-negative breast cancer, *met*. metastasis

### Detection of copy number alterations in CTCs

To learn more about the chromosomal aberrations present in BCBM and their changes during the metastasis process, we analyzed the copy number alteration (CNA) profiles of CTCs as well as autologous tumor tissue.

**Fig. 3:**
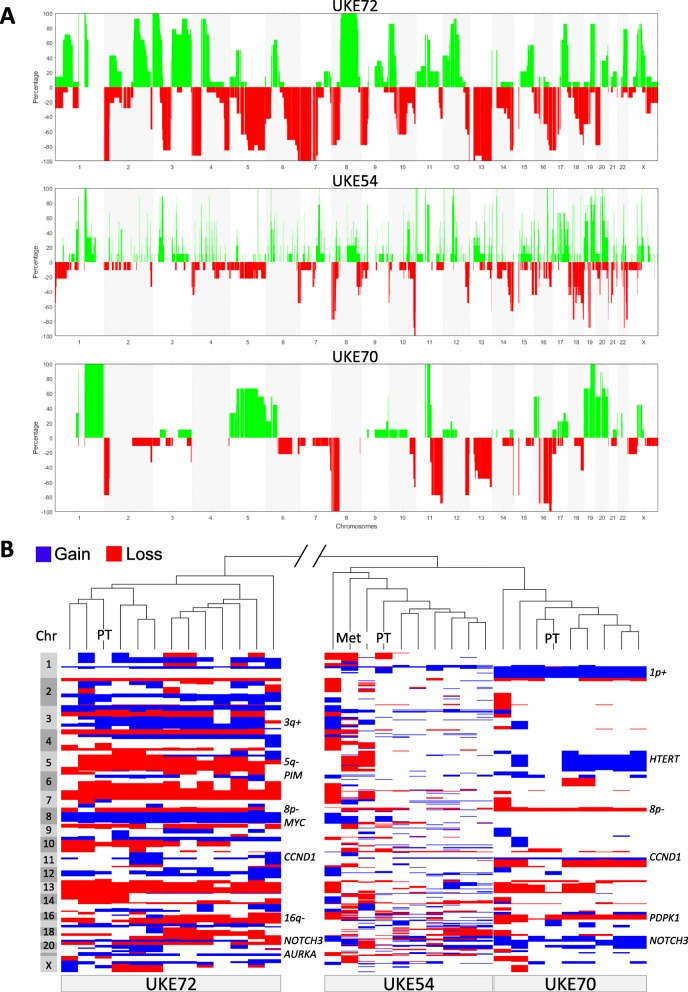
Copy number alterations. **a** Frequency plots of copy number gain (positive values, green) and loss (negative values, red) along the whole genome (*x*-axis) of tumor cells from three BCBM patients. **b** Unsupervised hierarchical clustering analysis of all individual CNA profiles of CTCs and tumor tissues of three BCBM patients, along with the corresponding heatmap showing copy number gain (blue) and loss (red) along the whole genome

CNA profiles were assessed in 28 CTCs from three different BCBM patients. From all three patients, the tissue of the corresponding primary breast tumor was available, as well as brain metastasis tissue from one patient. In two patients, no brain operation was performed. The CNA profiles of the single CTCs harbored typical aberrations corresponding to their primary tumor’s histological subtype. For instance, gain of chromosome 1p and loss of 16q are often observed in hormone receptor-positive tumors [19, 20] and could also be seen in the tumor cells of the hormone receptor-positive case UKE70 (Fig. 3a). This patient’s tumor cells also carried an amplification in each CTC of the 11q13.3 locus containing Cyclin D1 gene (*CCND1*), as well as gains of *NOTCH3* (19p13.2), HTERT (5p15.33), and *PDPK1* (16p13.3). In general, all eight CTCs were of high clonality showing very similar CNA profiles and thereby residing all together in unsupervised hierarchical clustering (Fig. 3b).

From case UKE72, a patient diagnosed with triple-negative breast cancer, 13 single CTCs and the primary tumor tissue were analyzed. The genomes of the investigated cells and tissue showed a large number of copy number aberrations, typically found in TNBC patients [21–23] and also described as a “sawtooth” profile [20]. These aberrations included gains of chromosomes implicated in metastatic breast cancer including 3q, 6p21.2 (*PIM1*), 8q22.1 (*CCNE2*), 8q4.21 (MYC), 11q13.3 (CCND1), 19p13.2 (*NOTCH3*), and 20q13.2 (*AURKA*) and losses of chromosomes 5q12-13 and 16q. All CTCs resided together along with the primary tumor when comparing the CNA profiles with the other samples (Fig. 3b).

A second case with TNBC, UKE54, all seven of the analyzed single CTCs showed a high number of small aberrations, being described as a “firestorm” profile [20]. Although with lower frequency, aberrations typical for triple-negative and basal-like breast cancer [22, 24] could be discerned in the CTCs as well as the corresponding primary tumor and brain metastases (Fig. 3a). Unsupervised hierarchical clustering analyses of all samples demonstrated also a high clonality within case UKE54 as all CTCs and tumor tissues resided together.

In summary, the CNA patterns showed a high clonality among different CTCs of the same patient and genomically resembling the primary breast tumors. In order to find potential brain metastasis-related aberrations, all samples were investigated on the most frequent CNAs. Only one region was gained in all samples located on chromosome 1q22-q23.2 containing among others the well-known gene *MUC1*. More interestingly, gain of chromosome 11p11.2 was seen in all but one sample. This genomic region contains the genes *AMBRA1*, *HARBI1*, *ATG13*, *ARHGAP1*, *ZNF408*, *F2*, *CKAP5*, *MiR5582*, *SNORD67*, *LRP4-AS1*, *LRP4*, and *C11orf49*.

### Mutation analysis in CTCs

In order to discover potential druggable targets, the NGS exome-seq data were investigated for mutations in known cancer-associated genes. To prevent the detection of false positives, mutation analysis was limited to mutations with a minimal coverage of six reads and reported in the COSMIC database only. After filtering, cancer-related mutations could be detected in 12/13 CTCs of case UKE72, 7/7 CTCs of case UKE54, and 7/8 CTCs of case UKE70 (Additional file 1: Data S1). The most frequent mutated gene was TP53, in which a mutation was detected in 50% of all CTCs. Other genes that were found to be mutated in at least 2 CTCs per patient were *ARID1A*, *CDH1*, and *TTN*. Genes that were mutated in 1 CTC in at least 2 patients were *RYR2*, *LRP2*, and *PI3KCA*. Although more cancer-related mutations were detected, we considered them as potentially false positive and therefore not report them. The most interesting target found was *ARID1A*, which is involved in chromatin remodeling and was found mutated (p.S2264* (stop); c.6791C>G) in CTCs of case UKE70. Therefore, ARID1A was investigated further in brain metastasis tissue.

### ARID1A expression in brain metastasis tissue

Analyzing our TMA, we classified ARID1A protein expression as negative, intermediate, or strong. Evaluable results were obtained from 95 brain metastases. Representative staining is shown in Additional file 2: Figure S1. 67.4% (64/95) of all brain metastases showed no ARID1A protein expression, whereas only 8.4% (8/95) of the samples showed a strong nuclear expression and 24.2% (23/95) had an intermediate expression. Negative expression of ARID1A was most commonly seen in TNBC brain metastases (82.9%, 29/35) compared to 51.3% (19/37) in ERBB2-positive and 71.4% (10/14) in hormone receptor-positive metastases (*p* = 0.017, *G* test). No other association between ARID1A expression in brain metastases and clinical status was found.

## Discussion

Breast cancer brain metastasis (BCBM) has a very poor prognosis, and reliable blood-based biomarkers are urgently needed to improve treatment options [3]. Detection of EpCAM-positive CTCs by the FDA cleared CellSearch System has already been shown to have a prognostic impact in many tumor entities [25, 26]. Large meta-analyses on the role of CTCs detected CellSearch System in both early- and late-stage breast cancer patients have clearly shown their predictive power among breast cancer patients. Here we used two different methods for CTC enrichment in BCBM patients’ blood: dependent and independent of EpCAM, as previous reports have indicated that brain metastatic patients might have many EpCAM-negative CTCs [27–29]. Indeed, we could observe that in our BCBM study population only 20.5% of the patients had CTCs (≥ 5 CTCs/7.5 ml blood) when analyzed by the CellSearch System using EpCAM as an enrichment marker, whereas the EpCAM-independent method detected CTCs in 32% of patients. Combining both methods for CTC detection, 63.6% of the patients showed CTCs with an overlap of only 27.3%. Among hormone receptor-positive patients, very few cases were CTC positive whereas in TNBC patients the EpCAM-independent enrichment resulted in a superior detection rate. Therefore, CTC assays based on epithelial surface markers such as the CellSearch System might not be detecting CTC populations of a mesenchymal character, a typical feature of TNBC. In general, the low CTC counts are a drawback in basic research or clinical tests and may be explained by the fact that the brain constitutes a unique microenvironment, with cells spreading to the brain having to go through certain steps of transformation in order to penetrate the blood-brain barrier. We have recently shown that also lung cancer patients with brain metastases have less CTCs when detected by the EpCAM-dependent CellSearch system [30]. Interestingly, we could show both in lung cancer as well as in this study that survival after brain metastasis diagnosis correlates with CTC detection when using EpCAM-dependent enrichment. This indicates that CTCs have a prognostic value in BCBM patients and regardless of the low counts can still be used as a prognostic marker for patient outcome.

Single-cell analysis enables characterization of tumor heterogeneity. For this reason, their characterization is thought to have a high potential for clinical impact. Here, we compared the CNA profiles of CTCs and those of corresponding tumor tissue. We performed CNA profiles from three patients, all corresponding primary breast tumors and one brain metastasis. We could show that corresponding CTCs resemble those of primary breast tumors, but identified alterations also in pathways known to be important in brain metastasis formation including notch (gain of *NOTCH3*) and PI3K (gain of *PDPK1*) pathways [29, 31, 32]. Interestingly, most of the CTCs within one patient showed a high clonality indicating that cells competent for brain metastases have undergone a strict clonal selection. High clonal restriction is also supported by the finding by Brastianos et al. in which they showed that different brain metastases in the same patients (also subsequential appearance) have remarkable similar genomes [33].

Mutation analysis showed alterations in cell cycle regulators such as *TP53*, *RB1*, and *CDKN2A*, as well as genes belonging to the PI3K pathway (*PTEN*, *PIK3CA*) and regulators of EMT (*CDH1*) and chromatin remodeling (*ARID1A*). Inactivating mutations of *ARID1A*, a subunit of the SWI/SNF chromatin remodeling complex, have been commonly reported in multiple human cancers, and especially in gynecological cancers [34]. Interestingly, *ARID1A* mutations frequently co-occur with *PIK3CA* or *PTEN* mutations in human tumors, and double mutations of *Arid1a* with *Pten* or *Pik3ca* result in ovarian tumor formation in mice [35, 36] suggesting a cooperative carcinogenic role of PI3K and chromatin remodeling pathways. As we and others have shown the importance of PI3K kinase pathway in brain metastases [7, 37], we analyzed here the expression pattern of ARID1A in brain metastases. We could show that 67% of all brain metastases have no ARID1A protein expression. The loss of ARID1A seems to be most commonly seen in TNBC brain metastases (83%) compared to 51% in ERBB2-enriched and 71% in HR-positive tumors. However, the occurrence of *ARID1A* mutations seems to be rare in triple-negative IDC-NST tumors and more frequently in metaplastic breast carcinoma (1 vs 11%) [38]. ARID1A expression did not correlate with any other clinicopathological factor, and thus, its role in brain metastases remains unclear. However, as depletion of ARID1A protein expression was found to significantly increase the sensitivity of cancer cells towards PI3K and AKT inhibitors, the expression of ARID1A could serve as a biomarker to predict the response for the inhibitors [39, 40]. Clearly, further studies need to be performed to validate these results.

## Conclusion

Our study shows the prognostic impact of CTC detection in BCBM patients. However, low detection rates highlight the challenges for the detection of CTCs in breast cancer patients with brain metastasis. Further investigation is required to identify driver-specific routes to dissemination to the brain, and implementation of large cohort studies is needed. Brain metastasis patients still have a very poor prognosis, and blood-based markers could generate a high impact on therapeutic management of these patients.

## Additional files

Additional file 1: ** Data S1.** Genes found to be mutated in CTCs using NGS exome-seq data. Below each case the frequency of affected genes is noted as well as for the complete cohort (bottom). (XLSX 9 kb)

Additional file 2: **Figure S1.** ARID1A staining of breast cancer brain metastasis tissue from a tissue microarray. (PPTX 789 kb)

## Data Availability

NGS data can be accessed via the European Nucleotide Archive (https://www.ebi.ac.uk/ena) under accession number EGAD00001005020.

## Abbreviations

BCBM: Breast cancer brain metastasis
CNA: Copy number alteration
CTC: Circulating tumor cell
NGS: Next-generation sequencing
TNBC: Triple-negative breast cancer
HR: Hormone receptor
