# Supplementary figures and images for: Clonality of circulating tumor cells in breast cancer brain metastasis patients
Source: Breast Cancer Res. 2019 Sep 3;21:101. doi: 10.1186/s13058-019-1184-2 (PMC6720990; doi:10.1186/s13058-019-1184-2)

## Slide 1
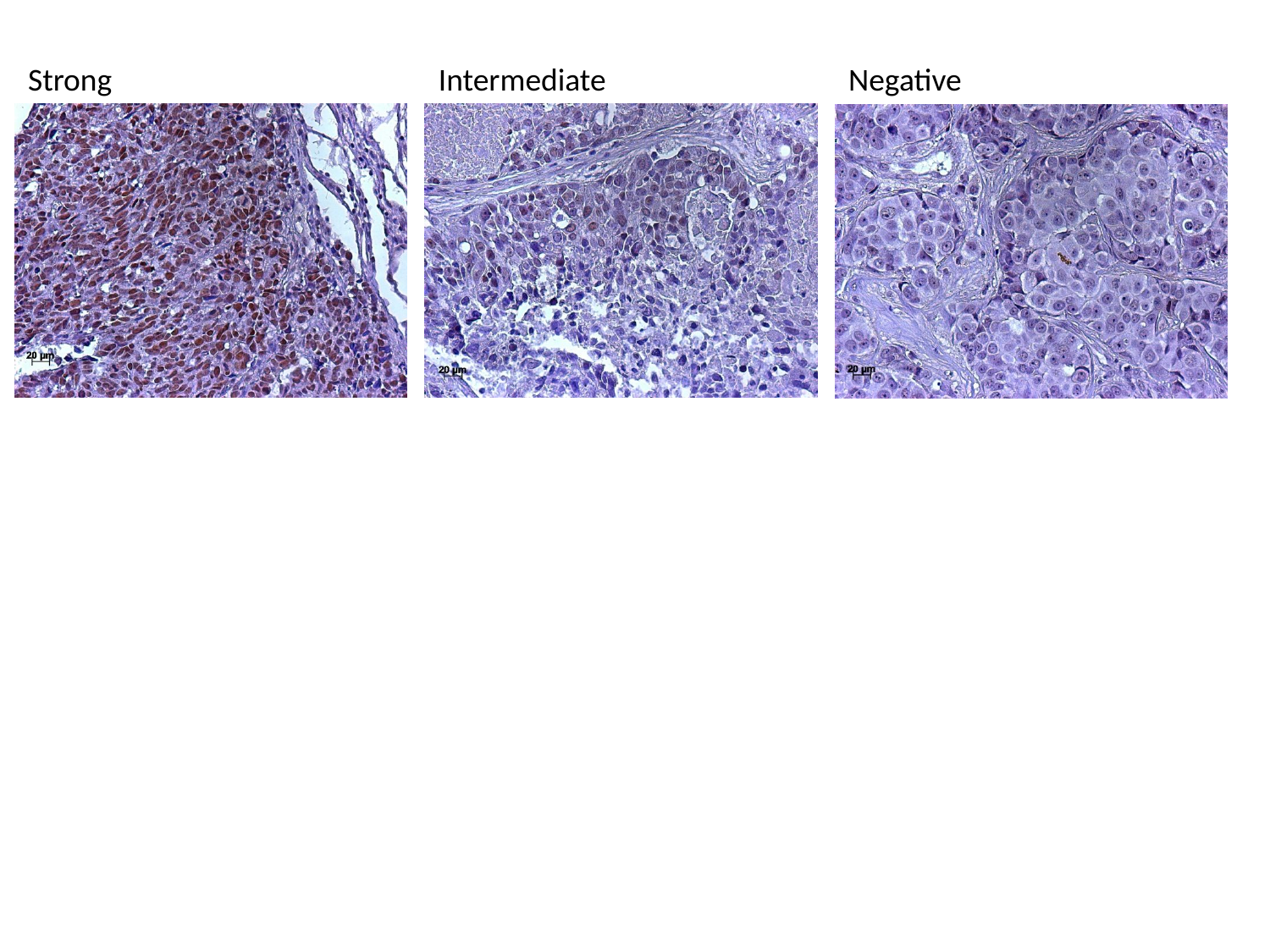

Strong
Intermediate
Negative

Supplement: Supplementary file 2 — Figure S1. ARID1A staining of breast cancer brain metastasis tissue from a tissue microarray. (PPTX 789 kb) [file 13058_2019_1184_MOESM2_ESM.pptx]
